# CD73^+^CD8^+^ T cells define a subset with anti-tumor potential in DLBCL patients

**DOI:** 10.3389/fmed.2025.1526772

**Published:** 2025-05-02

**Authors:** Lingyu Zhang, Rui Cheng, Zongbing Fan, Yunxiao Liu, Jie Huang, Jiabing Peng

**Affiliations:** Department of Pharmacy, The Fuyang Hospital of Anhui Medical University, Fuyang, Anhui, China

**Keywords:** CD8^+^ T cells, CD73, DLBCL, cytotoxicity, anti-tumor potential, immunotherapy

## Abstract

**Introduction:**

CD73, a recently discovered immune checkpoint, catalyzes the conversion of AMP to adenosine, thereby suppressing anti-tumor immune responses.CD8^+^ T cells play a critical role in the immune response against cancer, yet their functionality can be modulated by various factors within the tumor microenvironment. In this study, we focus on identifying and characterizing CD73^+^CD8^+^ T cells in the peripheral blood of patients with diffuse large B-cell lymphoma (DLBCL), aiming to elucidate their functional and phenotypic roles in tumor immunity.

**Methods:**

Using flow cytometry, we analyzed the expression of inhibitory receptors (e.g., PD-1, TIM-3) and activating markers (e.g., CD25, CD69) on CD73^+^CD8^+^ T cells compared to CD73^−^CD8^+^ T cells. *In vitro* functional assays were conducted to assess their cytotoxic activity against tumor cells, including cytokine production and tumor cell killing capacity.

**Results:**

CD73^+^CD8^+^ T cells exhibited a distinct immunophenotypic profile, characterized by reduced expression of inhibitory receptors and enhanced cytotoxic activity compared to their CD73^−^ counterparts. These cells demonstrated higher levels of effector molecules (e.g., IFN-γ, TNF-α) and lower exhausted markers. The findings suggest that CD73^+^CD8^+^ T cells may retain stronger anti-tumor potential.

**Discussion:**

This study highlights CD73^+^CD8^+^ T cells as a unique functional subset with potential therapeutic relevance in DLBCL. Their reduced exhaustion and heightened cytotoxicity position them as promising targets for immunotherapy strategies. However, the dual role of CD73 in adenosine-mediated immunosuppression warrants further investigation to reconcile its pro-tumorigenic effects with the observed anti-tumor activity of CD73^+^CD8^+^ T cells. Our findings deepen the understanding of CD8^+^ T cell heterogeneity in DLBCL and emphasize the need for mechanistic studies to explore CD73’s context-dependent functions.

## 1 Introduction

Diffuse large B-cell lymphoma (DLBCL) constitutes the most prevalent subtype of non-Hodgkin’s lymphoma (NHL), representing 30–40% of lymphoid malignancies (1). The current standard first-line treatment regimen, R-CHOP (rituximab, cyclophosphamide, adriamycin, vincristine, and prednisone), demonstrates efficacy in over 60% of patients (2, 3). However, approximately 30% of patients experience relapse with a poor prognosis, and a substantial number succumb to refractory lymphoma (3, 4).

Anti-tumor immune cells in peripheral blood predominantly consist of T cells, NK cells, DC cells, macrophages, and B cells, with CD8^+^ T cells being the primary drivers of the anti-tumor immune response (5). CD8^+^ T cells are typically viewed as a homogeneous population, recognized for producing substantial amounts of IFN-*γ*, TNF-*α*, and the protease granzyme B (6). However, within the tumor microenvironment (TME), CD8^+^ T cells are subjected to chronic antigen exposure and persistent T-cell receptor (TCR) stimulation (7–9). These CD8^+^ T cells become exhausted, characterized by the expression of inhibitory molecules such as PD-1, TIM-3, and TOX, resulting in the loss of cytotoxic effector functions (10–12).

CD73, also known as ecto-5′-nucleotidase, is the rate-limiting enzyme responsible for catalyzing the conversion of extracellular AMP into adenosine (13). CD73 is extensively expressed in malignant cells and facilitates adenosine production, which accumulates in the tumor microenvironment (14, 15), impeding immune cell infiltration and suppressing their function (16). Adenosine typically inhibits T-cell activation and proliferation by binding to the A2A receptor on effector T cells (17–19). The blockade of CD73 had no impact on primary tumor growth in T-cell-deficient mice, suggesting that CD73 promotes tumor growth in a T-cell-dependent manner, without influencing NK cells (20, 21). Moreover, overexpression of CD73 in mouse PDAC cells has been associated with enhanced tumor progression and immune evasion. In contrast, the CD73 inhibitor AB860 has been shown to reduce tumor burden and extend survival when administered, with its effects reliant on CD8^+^ T cells to elicit anti-tumor responses (22). Thus, CD73 has been proposed as a suppressor receptor for CD8^+^ T cells (23). However, the functional characteristics of CD73^+^CD8^+^ T cells across different contexts remain unclear.

In this study, we sought to characterize the functional characteristics and therapeutic potential of CD73^+^CD8^+^ T cells within the peripheral blood compartment of patients with DLBCL. We found that CD73 expression was higher in CD8^+^ T cells from healthy donors (HDs) than from DLBCL patients. CD73 was more expressed in naïve T cells and least expressed in terminally differentiated effector cells. We then examined inhibitory and activating receptors on the surface of CD8^+^ T cells and found that CD73^+^CD8^+^ T cells expressed fewer inhibitory receptors and more activation indicators compared to CD73^−^CD8^+^ T cells. Next, we stimulated CD8^+^ T cells with anti-CD3/CD28 and found that the CD73^+^CD8^+^ T cell subset was more capable of secreting cytokines than the CD73^−^CD8 + T cell subset. Finally, we co-cultured CD73^+^CD8^+^ T cells or CD73^−^CD8^+^ T cells with DLBCL cell lines (SU-DHL-6, OCI-Ly3) *in vitro* and found more apoptosis in DLBCL cell lines co-cultured with CD73^+^CD8^+^ T cells. These data demonstrate that CD73^+^CD8^+^ T cells have stronger cytotoxicity compared with their CD73^−^ counterparts, and might be a superior choice for CD8^+^ T cells cell-mediated immunotherapy.

## 2 Materials and methods

### 2.1 Patients

Peripheral blood samples of 20 HDs and 26 patients with DLBCL were from the Fuyang Hospital of Anhui Medical University. None of DLBCL patients selected for this study had received R-CHOP or chemotherapy. The study protocol was approved by the Medical Ethics Committee of the Fuyang Hospital of Anhui Medical University (Anhui, China) and was carried out in accordance with the Declaration of Helsinki 1964 and its later amendments. Written informed consent was obtained from all study participants.

### 2.2 Isolation of peripheral blood mononuclear cells

Peripheral blood mononuclear cells (PBMCs) were isolated from peripheral blood by gradient centrifugation with Ficoll (Solarbio, Beijing, China). Briefly, fresh peripheral blood was diluted with 1 × phosphate-buffered saline (PBS), transferred gently to Ficoll, and then centrifuged at 500 × g for 30 min at room temperature. After centrifugation, the intermediate layer was collected and washed with 1 × PBS. Lastly, PBMCs were counted in a cell counter (RWD, Shenzhen, China).

### 2.3 CD73^+^CD8^+^ T cell and CD73^−^CD8^+^ T cell isolation

CD8^+^ T cells were isolated with CD8^+^ T cell isolation kit (Biolegend, 480,011, San Diego, CA, United States) from PBMCs. Using the LS magnetizing column, usually 90% pure populations of CD8^+^ T cells are obtained. Subsequently, we used anti-PE MicroBeads (MACS™, 130–048-801, Bergisch Gladbach, Germany) to isolate CD73^+^CD8^+^ T cells and CD73^−^CD8^+^ T cells. The MS magnetization column was populated with CD73^+^CD8^+^ T cells, while the droplets contained CD73^−^CD8^+^ T cells.

### 2.4 Cell culture and stimulation of PBMCs

The DLBCL cell lines SU-DHL-6 and OCI-Ly3 were sourced from the American Type Culture Collection. All cells were kept in an incubator at 37°C with 5% CO2. SU-DHL-6 and OCI-Ly3 were cultured in RPMI-1640 (Gibco, Thornton, Australia) supplemented with 10% fetal bovine serum (Gibco) and 1% penicillin and streptomycin (Gibco) at 37°C and 5% CO2. CD8^+^ T cells were cultured in RPMI-1640 medium supplemented with 10% fetal calf serum (FCS), L-glutamine, penicillin/streptomycin, non-essential amino acids, sodium pyruvate, HEPES, *β*-2-mercaptoethanol at 37°C in a 5% CO2 atmosphere. To assess intracellular cytokine production, PBMCs were stimulated with anti-CD3 (2 μg/ mL) and anti-CD28 (1 μg/mL) (Biolegend) for 48 h. Brefeldin A (5 μg/mL) was added 4 h before collecting cells.

In order to assess the effector function of CD8^+^ T cells, inoculate logarithmic growth SU-DHL-6 or OCI-Ly3 cells into a 96-well U-bottom plate, with 1 × 10^5^ cells per well. CellTrace Violet (Thermo Fisher Scientific, Waltham, MA, United States) was added at a final concentration of 5 μM and the unbound dye was washed away after 30 min of incubation away from light. After that, the CD73^+^CD8^+^ T cells and CD73^−^CD8^+^ T cells obtained by MACS were added to 96-well plate with 1 × 10^5^ cells per well. The final volume of each well was adjusted to 200 μL. All wells were mixed thoroughly and cultured in a CO2 incubator at 37°C for approximately 72 h. After incubation, 7-AAD (BioLegend) was added and the samples were analyzed directly by flow cytometry.

### 2.5 Flow cytometry

Single-cell suspensions of PBMCs were stained with the fluorescently-labeled antibodies (all agents were from Biolegend), including APC/Cy7 anti-human CD3 (clone OKT3); PE/ Cy7 anti-human CD8 (clone RPA-T8); APC anti-human CD14 (clone HCD14); PE anti-human CD11c (clone Bu15); Brilliant Violet 421™ anti-human CD56 (clone HCD56); Brilliant Violet 605™ anti-human HLA-DR (clone QA19A44); Brilliant Violet 605™ anti-human CD366 (Tim-3) (clone F38-2E2); Brilliant Violet 421™ anti-human CD279 (PD-1) (clone EH12.2H7); FITC anti-human CD244 (2B4) (clone 2–69); PerCP/Cyanine5.5 anti-human TIGIT (clone A15153G); APC anti-human CD39 (clone A1); Brilliant Violet 421™ anti-human CD197 (CCR7) (clone G043H7); PerCP/Cyanine5.5 anti-human CD45RA (clone HI100); FITC anti-human CD69 (clone FN50); APC anti-human CD25 (clone BC96). Then, cells underwent intracellular staining with APC anti-human tumor necrosis factor (TNF)-*α* (clone MAb11); PE/Cy7 anti-human-IFN-*γ* (clone B27); Brilliant Violet 421™ anti-human/mouse Granzyme B Recombinant (clone QA18A28) using an intracellular fixation and permeabilization buffer set according to manufacturer (eBioscience, San Diego, CA, United States) instructions. Data were acquired using a flow cytometer (Beckman Coulter; Brea, CA, United States) and analyzed using FlowJo 10.8.1 (Tree Star, Ashland, OR, United States).

### 2.6 Cytotoxicity assay

The cytotoxicity of CD73^−^CD8^+^ T cells and CD73^+^CD8^+^ T cells was evaluated using CCK-8 assay in SU-DHL-6 cells or OCI-Ly3 cells. Effector cells (1 × 10^5^) were placed in a 96-well U-bottom plate and then mixed with various numbers of target cells (1:1, 1:2, and 1:4 effector-to-target (E: T) ratios). The plates were centrifuged at 1,500 rpm for 3 min and then incubated for 72 h at 37°C in a 5% CO2 incubator. After that, 10 μL of CCK-8 (Biosharp, Hefei, China) solution was added to each well and incubated for 2 h. The absorbance of effector cells wells (A_e_), sample wells (A_s_) and target cells wells (A_t_) were measured with a microplate reader at a test wavelength of 450 nm and a reference wavelength of 690 nm. Percentage specific killing was determined using the formula: 1-(A_s_-A_e_)/A_t_ × 100%. All experiments were repeated in triplicate.

### 2.7 Statistical analyses

Statistical analyses were undertaken using Prism 9.5.0 (GraphPad, San Diego, CA, United States). A paired or unpaired Student’s *t*-test was used to determine significance. Data are the mean ± standard deviation from at least five independent experiments. *p* < 0.05 was considered significant.

## 3 Results

### 3.1 CD8^+^ T cells in the peripheral blood of patients with DLBCL exhibit reduced expression of CD73

**Figure 1:**
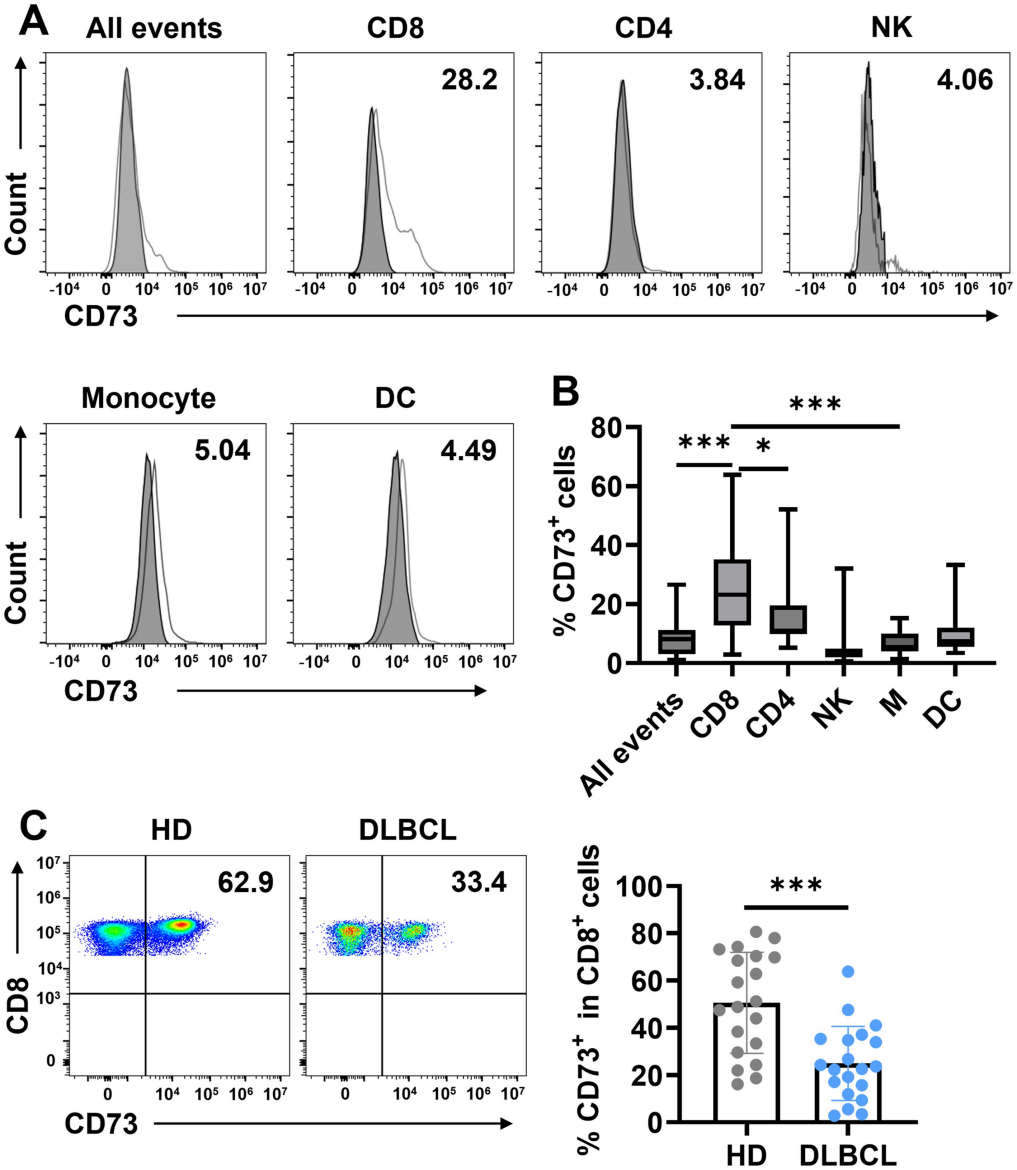
CD73 is downregulated on CD8^+^ T cells in patients with DLBCL compared with HDs. **(A,B)** The expression of CD73 on all events, T cells (CD4^+^ T cells, CD8^+^ T cells), natural killer cells (NK), and myeloid cells (monocytes and dendritic cells) was assessed by flow cytometry. Representative flow data **(A)** and summary plot **(B)** of CD73. Data are shown as means ± standard deviation (SD). *p* values were obtained by the unpaired t test or Mann–Whitney test. *p < 0.05, ***p < 0.001. **(C)** Proportion of CD73^+^CD8^+^ T cells in total CD8^+^ T cells in HDs (*n* = 20) or patients with DLBCL (*n* = 20), each dot indicates one patient. Data are shown as means ± SD. *p* values were obtained by two-tailed unpaired *t* test. *p < 0.05, ***p < 0.001.

CD73 is generally considered an unfavorable prognostic marker in tumors. To determine which cells predominantly express CD73 in the peripheral blood of DLBCL patients, we utilized flow cytometry to analyze all events (live cells), T cells (CD4^+^ T cells, CD8^+^ T cells), natural killer cells (NK), and myeloid cells (monocytes and dendritic cells) in peripheral blood (Supplementary Figure S1). CD73 averages about 10% of peripheral blood in all events. And CD73 expression was the highest in CD8^+^ T cells, at approximately 25%, followed by CD4^+^ T cells and NK cells, each around 10% (Figures 1A,B). The lowest expression levels were observed in DCs and monocytes, at about 5% (Figures 1A,B). The results revealed that CD73 expression is reduced in CD8^+^ cells in the peripheral blood of DLBCL patients (Figure 1C). This data indicates that CD8^+^ T cells show the highest CD73 expression among immune cells, with this expression decreasing during DLBCL progression.

### 3.2 CD73 is less expressed on terminally differentiated effector cells (T_EMRA_)

**Figure 2:**
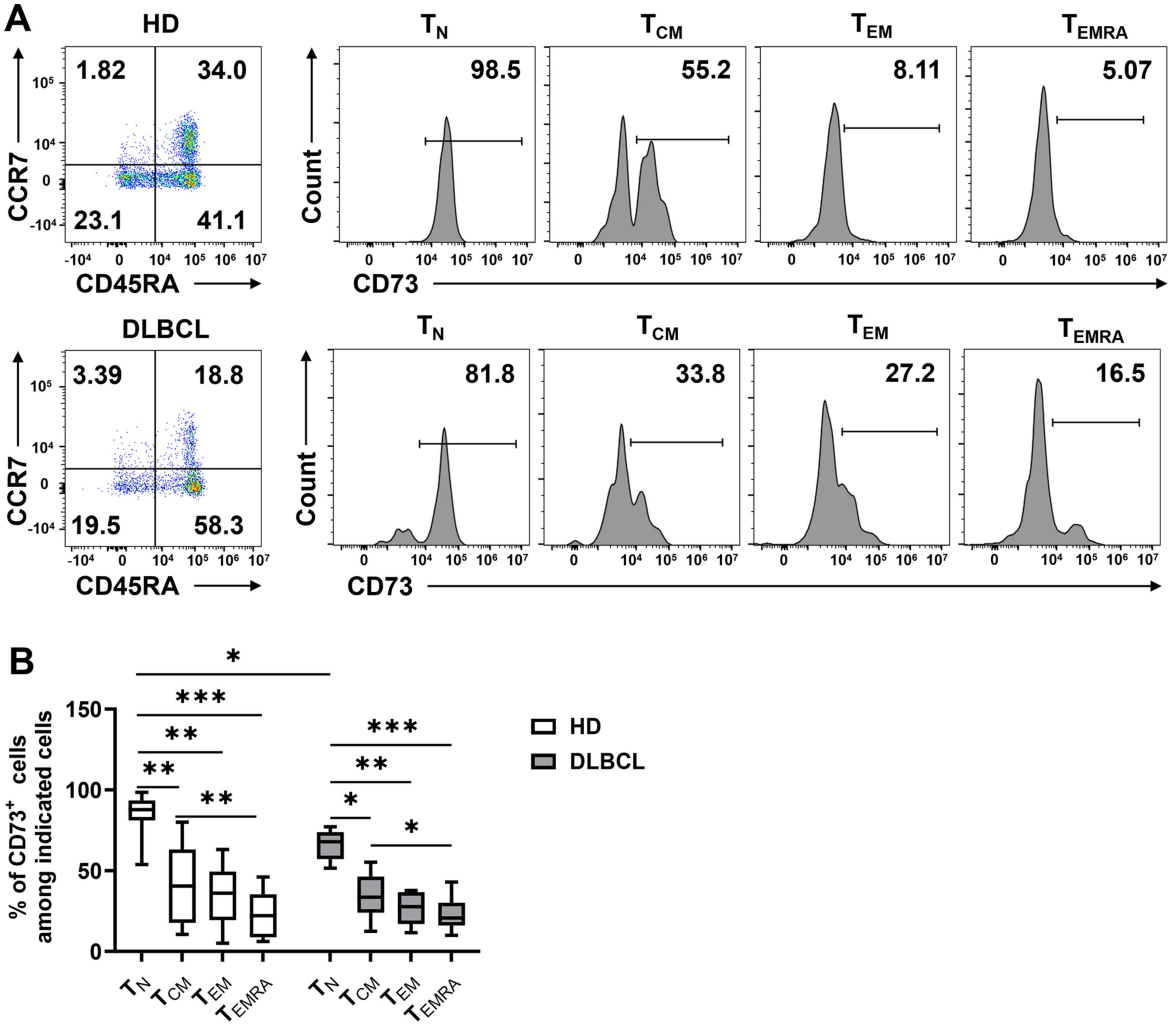
CD73 is less expressed on T_EMRA_. Expression of CD73 among each subset (T_N_, T_CM_, T_EM_, and T_EMRA_) of CD8 T cells was analyzed. Representative flow data **(A)** and plots **(B)** of percentage of CD73 expression among each subset of CD8^+^ T cells from HDs (*n* = 10) or DLBCL patients (*n* = 10) are shown. Data are shown as means ± SD. *p* values were obtained by Kruskal-Wallis test followed by Dunn’s multiple comparisons test, *p < 0.05; **p < 0.001; ***p < 0.001.

Next, we examined the phenotypic characteristics of CD73-expressing CD8^+^ T cells. Based on the expression of CD45RA and CCR7, T cells are categorized into four subsets: naïve T cells (T_N_; CCR7^+^CD45RA^+^), central memory T cells (T_CM_; CCR7^+^CD45RA^−^), effector memory T cells (T_EM_; CCR7^−^CD45RA^−^), and terminally differentiated effector cells (T_EMRA_; CCR7^−^CD45RA^+^). We assessed CD73 expression across these subsets and found that it was significantly higher on T_N_ cells compared to T_CM_, T_EM_, and T_EMRA_ cells, indicating that CD73 is downregulated in CD8^+^ cells following antigenic stimulation, a pattern observed in both DLBCL patients and healthy individuals (Figures 2A,B). While no difference in CD73 expression on CD8^+^ T_EM_ and T_EMRA_ cells was noted between healthy controls and DLBCL patients, the CD8^+^ T_N_ subset in DLBCL patients showed lower CD73 expression than in healthy controls (Figure 2B). Notably, CD73 expression was lowest on T_EMRA_ cells, which are generally regarded as high cytotoxic T cells in the tumor microenvironment, exhibiting high cytotoxicity and pro-inflammatory capacity. Our data demonstrate that in DLBCL patients, CD73 is highly expressed on T_N_ cells and minimally expressed on T_EMRA_ CD8^+^ T cells, suggesting that the downregulation of CD73 is associated with the activation of an anti-tumor immune response by T cells.

### 3.3 CD73^+^CD8^+^ T cells express fewer inhibitory receptors and more activating markers

**Figure 3:**
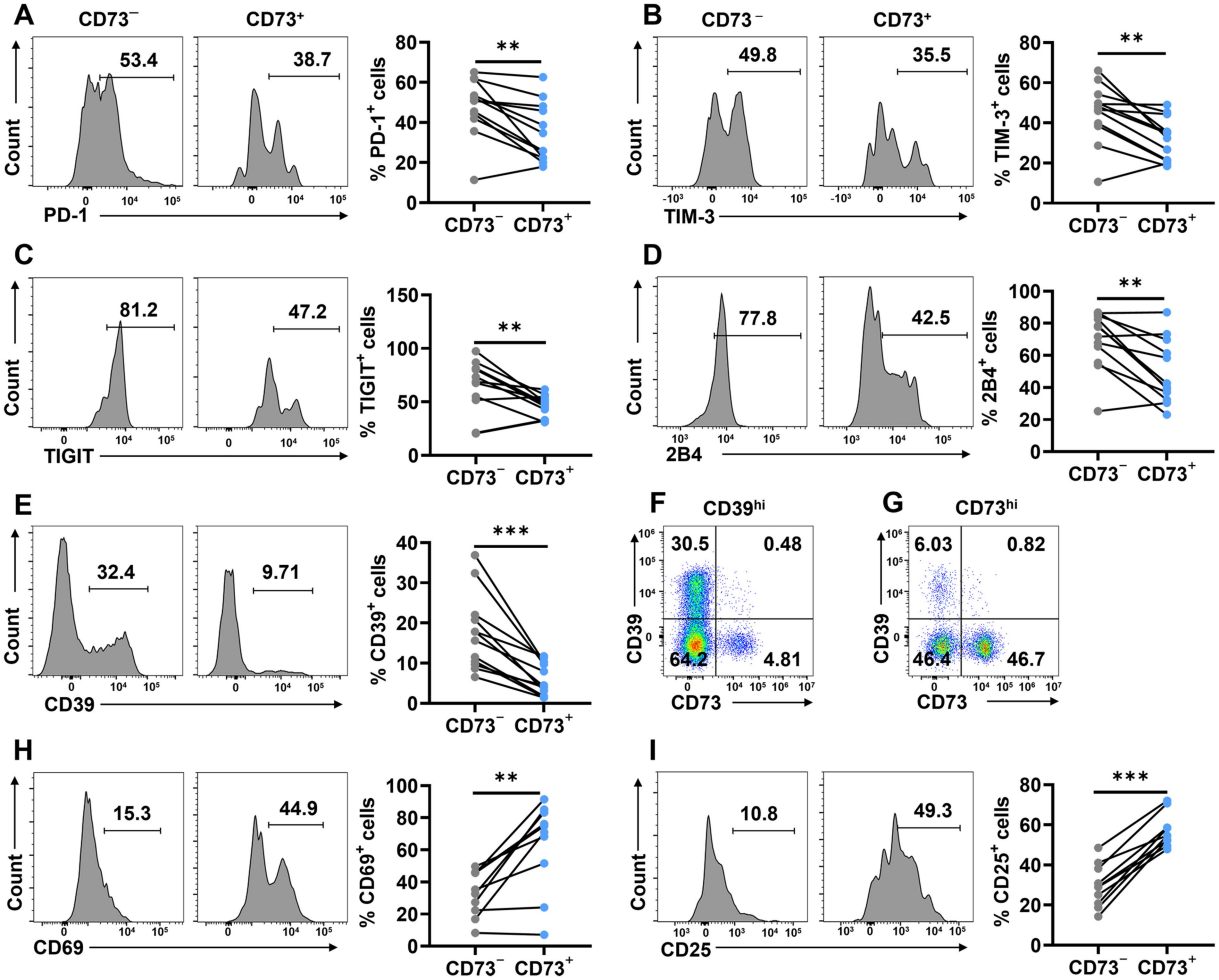
CD73^+^CD8^+^ T cells express fewer inhibitory and more activating receptors. **(A–E)** Proportion of PD-1^+^ **(A)**, TIM-3^+^ **(B)**, TIGIT^+^ **(C)**,2B4^+^ **(D)** and CD39^+^ **(E)** cells in CD73^+^CD8^+^ T cells and CD73^−^CD8^+^ T cells (*n* = 12). **(F)** Representative flow data of CD8^+^ T cells highly expressing CD39. **(G)** Representative flow data of CD8^+^ T cells highly expressing CD73. **(H–I)** Proportion of CD69^+^ **(H)**, and CD25^+^ **(I)** cells in CD73^+^CD8^+^ T cells and CD73^−^CD8^+^ T cells (*n* = 10). Data are the mean ± SD. A two-tailed paired t-test was used for statistical analyses, **p < 0.01, ***p < 0.001.

To further investigate whether the low expression of CD73 on CD8^+^ T cells in DLBCL patients is associated with status of exhaustion, we compared the expression of inhibitory receptors on CD73^−^ and CD73^+^ CD8^+^ T cells. We found that the expression of PD-1, TIM-3, TIGIT, and 2B4 was significantly higher on CD73^−^CD8^+^ T cells compared to CD73^+^CD8^+^ T cells (Figures 3A–D). Additionally, we examined CD39 and observed that its expression on CD73^−^CD8^+^ T cells was markedly greater than on CD73^+^CD8^+^ T cells, with minimal co-expression of CD39 and CD73 on CD8^+^ T cells (Figures 3E–G). The observation that CD73^−^CD8^+^ T cells express higher levels of inhibitory receptors could indicate that they are exhausted or dysfunctional, which might also contribute to anti-tumor immunity under certain conditions.

Given that CD73^+^CD8^+^ T cells exhibited fewer inhibitory receptors than CD73^−^CD8^+^ T cells, we proceeded to assess the activation status of CD73^+^CD8^+^ T cells by measuring CD69 and CD25. The proportion of CD69^+^ and CD25^+^ cells was higher in CD73^+^CD8^+^ T cells than in CD73^−^CD8^+^ T cells, indicating that CD73^+^CD8^+^ T cells are more activated compared to their CD73^−^CD8^+^ T cells counterparts (Figures 3H,I). Collectively, our findings suggest that CD73^+^CD8^+^ T cells express elevated levels of activating markers and reduced levels of inhibitory receptors, positioning them as highly activated and low exhausted T cells in DLBCL patients.

### 3.4 CD73^+^CD8^+^ T cells are high cytotoxic T cells

**Figure 4:**
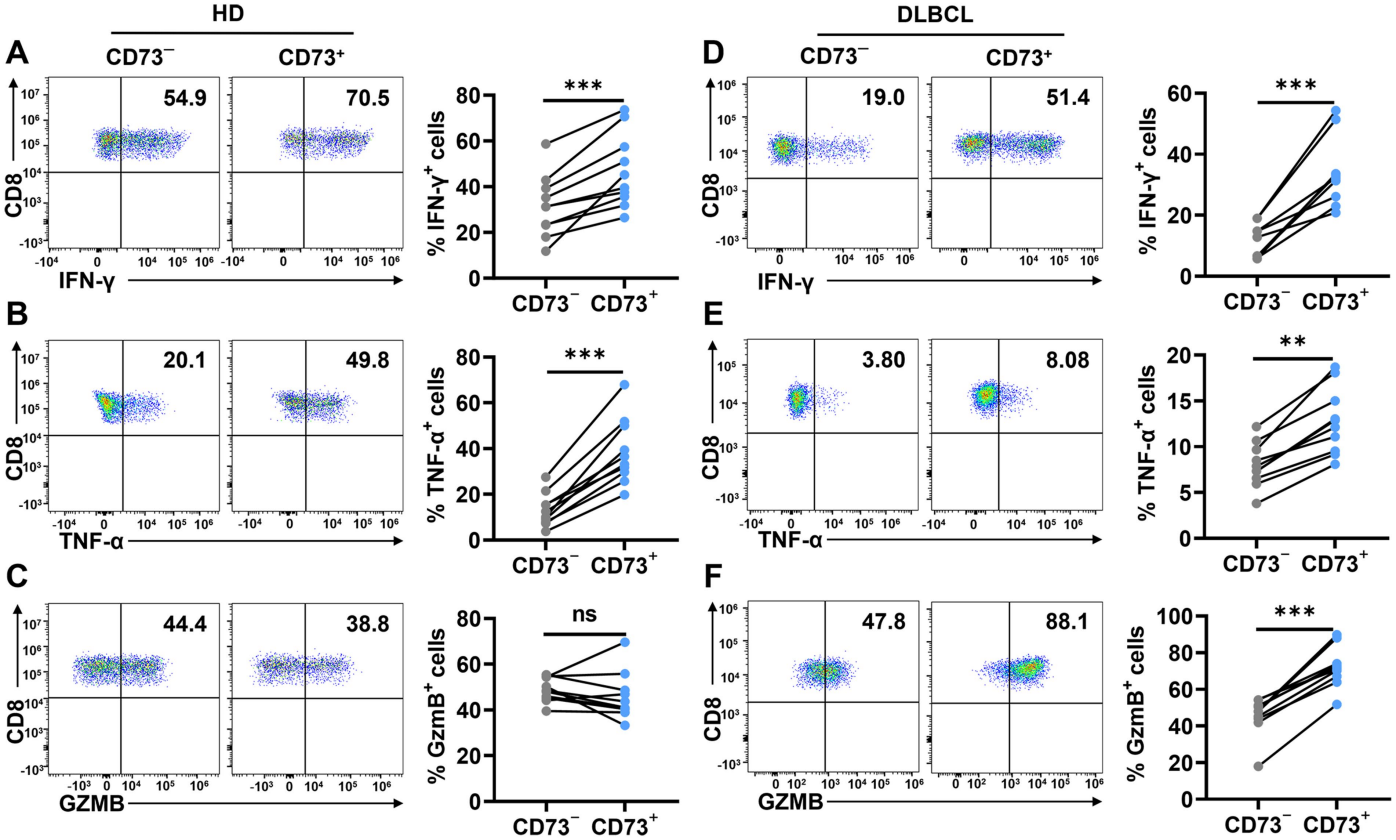
CD73^+^CD8^+^ T cells have higher effector functions. **(A–C)** Proportion of IFN-*γ*^+^ **(A)**, TNF-*α*^+^ **(B)** and GzmB^+^ **(C)** cells in CD73^+^CD8^+^ T cells and CD73^−^CD8^+^ T cells in HDs after stimulation with anti-CD3 (2 μg/ mL) and anti-CD28 (1 μg/mL) for 48 h (*n* = 10). **(D–F)** Proportion of IFN-γ^+^ **(D)**, TNF-α^+^ **(E)** and GzmB^+^ **(F)** cells in CD73^+^CD8^+^ T cells and CD73^−^CD8^+^ T cells in DLBCL after stimulation with anti-CD3 (2 μg/ mL) and anti-CD28 (1 μg/mL) for 48 h (*n* = 10). Data are the mean ± SD. A two-tailed paired t-test was used for statistical analyses, ns = non-significant, **p < 0.01, ***p < 0.001.

To assess CD73^+^CD8^+^ T cells’ function, we conducted *in vitro* assays stimulating CD8^+^ T cells with anti-CD3/CD28 to evaluate intracellular cytokine production in two CD8^+^ T cell subsets. In peripheral blood from healthy donors, the CD73^+^CD8^+^ T cell subset displayed higher proportions of IFN-*γ*^+^ and TNF-*α*^+^ cells compared to the CD73^−^CD8^+^ T cell subset, while Granzyme B^+^ (GzmB) cell proportions were comparable between both subsets (Figures 4A–C). Similarly, in DLBCL patients’ peripheral blood, CD73^+^CD8^+^ T cells expressed higher levels of IFN-γ, TNF-α, and GzmB than CD73^−^CD8^+^ T cells following anti-CD3/CD28 stimulation (Figures 4D–F). These findings indicate that CD73^+^CD8^+^ T cells possess enhanced functional capabilities, consistent with high cytotoxic T cells.

### 3.5 CD73^+^CD8^+^ T cells performing better anti-tumour effects

**Figure 5:**
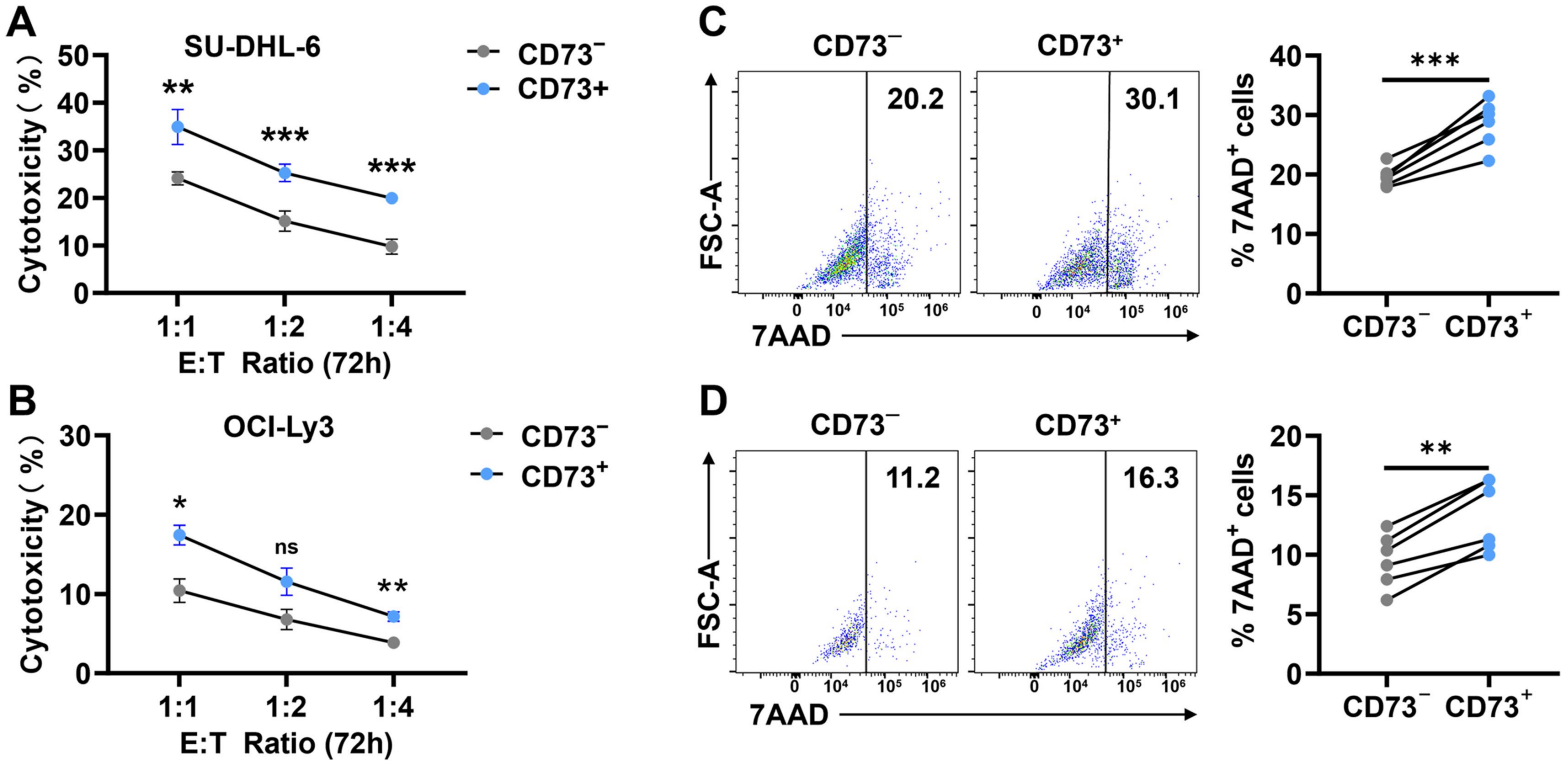
CD73^+^CD8^+^ T cells performing better anti-tumour effects. **(A,B)** Proportion of cytotoxicity after 72-h co-culture of CD73^+^CD8^+^ T cells or CD73^−^CD8^+^ T cells with SU-DHL-6 **(A)** (*n* = 4) or OCI-Ly3 **(B)** (*n* = 4) cells at different effector-to-target (E: T = 1:1, 1:2, and 1:4) ratios. Data are shown as mean ± standard error of the mean (SEM). *p* values were obtained by two-tailed unpaired t test was used for statistical analyses, ns = non-significant, *p < 0.05; **p < 0.001; ***p < 0.001. **(C,D)** Representative flow data and plots of percentage of apoptosis after 72-h co-culture of CD73^+^CD8^+^ T cells or CD73^−^CD8^+^ T cells with SU-DHL-6 **(C)** (*n* = 6) or OCI-Ly3 **(D)** (*n* = 6) cells. Data are the mean ± SD. p values were obtained by two-tailed paired t-test was used for statistical analyses, **p < 0.001; ***p < 0.001.

CD73^+^CD8^+^ T cells and CD73^−^CD8^+^ T cells from DLBCL patients were co-cultured with SU-DHL6 or OCI-LY3 cells to assess apoptosis. First, we performed CCK-8 assays to evaluate cytotoxicity after 72 h of co-culture at different effector-to-target (E: T = 1:1, 1:2, and 1:4) ratios (Figures 5A,B). Subsequently, flow cytometry was employed to assess SU-DHL6 (Figure 5C) or OCI-LY3 (Figure 5D) cells apoptosis at E: T = 1:1 after 72 h. The results revealed that CD73^+^CD8^+^ T cells significantly increased the overall apoptosis rate of SU-DHL-6 or OCI-LY3 cells. These findings suggest that CD73^+^CD8^+^ T cells in DLBCL patients exhibit enhanced anti-tumor effects compared to CD73^−^CD8^+^ T cells.

In conclusion, our findings suggested that in patients with DLBCL, CD73^−^CD8^+^ T cells expressed high levels of inhibitory receptors, were dysfunctional, consistent with exhaustion. And CD73^+^CD8^+^ T cells have higher potential anti-tumor activity compared to CD73^−^CD8^+^ T cells.

## 4 Discussion

CD8^+^ T cells play a crucial role in defending against various types of cancers and infections. They are heterogeneous and can be categorized into different subpopulations based on their phenotypic and functional properties. In this study, we report that CD73^+^CD8^+^ T cells, a subpopulation found in the peripheral blood of DLBCL patients, exhibit enhanced effector functions, characterized by lower expression of inhibitory receptors (PD-1, TIM-3, TIGIT, 2B4) and higher levels of activating markers (CD69, CD25). Furthermore, CD73^+^CD8^+^ T cells have been identified as high anti-tumor potential T cells. In contrast, the phenotype of CD73^−^CD8^+^ T cells resemble that of exhausted T cells as described in previous studies (11, 12, 23). Therefore, CD73^+^CD8^+^ T cells could be new choices for CD8^+^ T cell-based immunotherapy in DLBCL patients.

Several studies have confirmed that tumor cells can also express CD73, with its expression and activity closely linked to tumor invasion and metastasis (24, 25). Moreover, extracellular adenosine produced by CD73 in tumor cells is sufficient to mediate immune escape and promote tumor growth and metastasis. In contrast, other research has reported opposing findings, observing a correlation between high CD73 expression and favorable clinical outcomes (26–28). This discrepancy may arise from the former studies focusing on tumor and stromal cells, while the latter primarily examined CD73 expression on T cells.

Systemic therapy using blocking antibodies against CD73 has become a key approach in investigational trials targeting the CD73-adenosine pathway for cancer control. Previous studies have shown that CD73 inhibitors, when combined with immune checkpoint blockade (ICIs), effectively inhibit the progression of pancreatic (22), non-small cell lung cancer (NSCLC) (29), and breast cancers (30) in mouse models. However, we observed that CD73^−^CD8^+^ T cells expressed higher frequencies of negative receptors such as PD-1, TIM-3, and TIGIT, suggesting that the blockade of CD73 may upregulate inhibitory pathways in CD8^+^ T cells. Therefore, understanding the specific distribution pattern of CD73 across different tumor types is essential for optimizing the design of clinical studies targeting CD73 for cancer treatment.

Our findings align with those of Kong et al. (31) and Tóth et al. (32), indicating that CD73^+^CD8^+^ T cells exhibit reduced PD-1 expression, contrasting with the exhausted T cell phenotype. However, it has also been reported that CD73^+^CD8^+^ T cells are paracrine T cells lacking tumor antigen specificity within the tumor microenvironment of head and neck cancer patients (33). Unfortunately, a notable limitation of this study lies in its restriction to peripheral blood samples from DLBCL patients. While our flow cytometry-based phenotypic and functional characterization of CD73^+^CD8^+^ T cells provides initial insights, this methodological scope precludes comprehensive mechanistic interrogation of tumor-specific immune responses. Future investigations will require integrated multi-omics approaches (e.g., transcriptomics, proteomics, metabolomics) coupled with tumor-infiltrating lymphocyte profiling to establish microenvironmental congruence between circulating and intertumoral T cell populations.

In conclusion, our findings reveal the existence of a population of CD73^+^CD8^+^ T cells demonstrating enhanced effector functions coupled with reduced expression of exhaustion phenotypes, indicative of heightened anti-tumor potential. These findings may deepen our understanding of human CD8^+^ T cell heterogeneity, and improve CD8^+^ T cell-based immunotherapy.

## Data Availability

The original contributions presented in the study are included in the article/Supplementary material, further inquiries can be directed to the corresponding author.

## Supplementary material

Supplementary Figure S1 The flow cytometric gating strategy for analyzing the proportions of live cells, CD4^+^ T cells, CD8^+^ T cells, NK cells, monocytes and DC.

## References

[1] Sassolas B Labouche A Guillet G. Herpes simplex virus infection associated with bullous pemphigoid. Br J Dermatol. (1993) 128:358–9. doi: 10.1111/j.1365-2133.1993.tb00187.x, PMID: 8471526

[2] Li S Young KH Medeiros LJ. Diffuse large B-cell lymphoma. Pathology. (2018) 50:74–87. doi: 10.1016/j.pathol.2017.09.006, PMID: 29167021

[3] Crump M Neelapu SS Farooq U van den Neste E Kuruvilla J Westin J. Outcomes in refractory diffuse large B-cell lymphoma: results from the international SCHOLAR-1 study. Blood. (2017) 130:1800–8. doi: 10.1182/blood-2017-03-769620, PMID: 28774879 PMC5649550

[4] Carpio C Bouabdallah R Ysebaert L Sancho JM Salles G Cordoba R. Avadomide monotherapy in relapsed/refractory DLBCL: safety, efficacy, and a predictive gene classifier. Blood. (2020) 135:996–1007. doi: 10.1182/blood.2019002395, PMID: 31977002 PMC7099331

[5] Philip M Schietinger A. CD8+ T cell differentiation and dysfunction in cancer. Nat Rev Immunol. (2022) 22:209–23. doi: 10.1038/s41577-021-00574-3, PMID: 34253904 PMC9792152

[6] Sinkarevs S Strumfs B Volkova S Strumfa I. Tumour microenvironment: the general principles of pathogenesis and implications in diffuse large B cell lymphoma. Cells. (2024) 13:1057. doi: 10.3390/cells13121057, PMID: 38920685 PMC11201569

[7] Utzschneider DT Alfei F Roelli P Barras D Chennupati V Darbre S. High antigen levels induce an exhausted phenotype in a chronic infection without impairing T cell expansion and survival. J Exp Med. (2016) 213:1819–34. doi: 10.1084/jem.20150598, PMID: 27455951 PMC4995073

[8] Scott AC Dündar F Zumbo P Chandran SS Klebanoff CA Shakiba M. TOX is a critical regulator of tumour-specific T cell differentiation. Nature. (2019) 571:270–4. doi: 10.1038/s41586-019-1324-y, PMID: 31207604 PMC7698992

[9] Oliveira G Stromhaug K Klaeger S Kula T Frederick DT le PM. Phenotype, specificity and avidity of antitumour CD8+ T cells in melanoma. Nature. (2021) 596:119–25. doi: 10.1038/s41586-021-03704-y, PMID: 34290406 PMC9187974

[10] Schietinger A Philip M Krisnawan VE Chiu EY Delrow JJ Basom RS. Tumor-specific T cell dysfunction is a dynamic antigen-driven differentiation program initiated early during tumorigenesis. Immunity. (2016) 45:389–401. doi: 10.1016/j.immuni.2016.07.011, PMID: 27521269 PMC5119632

[11] Pauken KE Sammons MA Odorizzi PM Manne S Godec J Khan O. Epigenetic stability of exhausted T cells limits durability of reinvigoration by PD-1 blockade. Science (New York, NY). (2016) 354:1160–5. doi: 10.1126/science.aaf2807 PMC5484795 27789795

[12] Khan O Giles JR McDonald S Manne S Ngiow SF Patel KP. TOX transcriptionally and epigenetically programs CD8+ T cell exhaustion. Nature. (2019) 571:211–8. doi: 10.1038/s41586-019-1325-x, PMID: 31207603 PMC6713202

[13] Roh M Wainwright DA Wu JD Wan Y Zhang B. Targeting CD73 to augment cancer immunotherapy. Curr Opin Pharmacol. (2020) 53:66–76. doi: 10.1016/j.coph.2020.07.001, PMID: 32777746 PMC7669683

[14] Busse M Vaupel P. Accumulation of purine catabolites in solid tumors exposed to therapeutic hyperthermia. Experientia. (1996) 52:469–73. doi: 10.1007/BF01919318, PMID: 8641385

[15] Blay J White TD Hoskin DW. The extracellular fluid of solid carcinomas contains immunosuppressive concentrations of adenosine. Cancer Res. (1997) 57:2602–5. PMID: 9205063

[16] Xia C Yin S To KKW Fu L. CD39/CD73/A2AR pathway and cancer immunotherapy. Mol Cancer. (2023) 22:44. doi: 10.1186/s12943-023-01733-x, PMID: 36859386 PMC9979453

[17] Stagg J Smyth MJ. Extracellular adenosine triphosphate and adenosine in cancer. Oncogene. (2010) 29:5346–58. doi: 10.1038/onc.2010.292, PMID: 20661219

[18] Ohta A. A metabolic immune checkpoint: adenosine in tumor microenvironment. Front Immunol. (2016) 7:109. doi: 10.3389/fimmu.2016.00109 27066002 PMC4809887

[19] Ohta A Gorelik E Prasad SJ Ronchese F Lukashev D Wong MKK. A2A adenosine receptor protects tumors from antitumor T cells. Proc Natl Acad Sci USA. (2006) 103:13132–7. doi: 10.1073/pnas.0605251103, PMID: 16916931 PMC1559765

[20] Jin D Fan J Wang L Thompson LF Liu A Daniel BJ. CD73 on tumor cells impairs antitumor T-cell responses: a novel mechanism of tumor-induced immune suppression. Cancer Res. (2010) 70:2245–55. doi: 10.1158/0008-5472.CAN-09-3109, PMID: 20179192 PMC2883609

[21] Stagg J Divisekera U McLaughlin N Sharkey J Pommey S Denoyer D. Anti-CD73 antibody therapy inhibits breast tumor growth and metastasis. Proc Natl Acad Sci USA. (2010) 107:1547–52. doi: 10.1073/pnas.0908801107, PMID: 20080644 PMC2824381

[22] Chen Q Yin H He J Xie Y Wang W Xu H. Tumor microenvironment responsive CD8+ T cells and myeloid-derived suppressor cells to trigger CD73 inhibitor AB680-based synergistic therapy for pancreatic Cancer. Adv Sci. (2023) 10:e2302498. doi: 10.1002/advs.202302498 PMC10667825 37867243

[23] Deng W-W Li YC Ma SR Mao L Yu GT Bu LL. Specific blockade CD73 alters the "exhausted" phenotype of T cells in head and neck squamous cell carcinoma. Int J Cancer. (2018) 143:1494–504. doi: 10.1002/ijc.31534, PMID: 29663369 PMC11523565

[24] Zhang B. CD73: a novel target for cancer immunotherapy. Cancer Res. (2010) 70:6407–11. doi: 10.1158/0008-5472.CAN-10-1544, PMID: 20682793 PMC2922475

[25] Chen S Wainwright DA Wu JD Wan Y Matei DE Zhang Y. CD73: an emerging checkpoint for cancer immunotherapy. Immunotherapy. (2019) 11:983–97. doi: 10.2217/imt-2018-0200, PMID: 31223045 PMC6609898

[26] Bowser JL Blackburn MR Shipley GL Molina JG Dunner K Jr Broaddus RR. Loss of CD73-mediated actin polymerization promotes endometrial tumor progression. J Clin Invest. (2016) 126:220–38. doi: 10.1172/JCI79380, PMID: 26642367 PMC4701552

[27] Oh HK Sin JI Choi J Park SH Lee TS Choi YS. Overexpression of CD73 in epithelial ovarian carcinoma is associated with better prognosis, lower stage, better differentiation and lower regulatory T cell infiltration. J Gynecol Oncol. (2012) 23:274–81. doi: 10.3802/jgo.2012.23.4.274, PMID: 23094131 PMC3469863

[28] Wettstein MS Buser L Hermanns T Roudnicky F Eberli D Baumeister P. CD73 predicts favorable prognosis in patients with nonmuscle-invasive urothelial bladder Cancer. Dis Markers. (2015) 2015:785461. doi: 10.1155/2015/785461 26543299 PMC4620269

[29] Tu E McGlinchey K Wang J Martin P Ching SLK Floc’h N. Anti-PD-L1 and anti-CD73 combination therapy promotes T cell response to EGFR-mutated NSCLC. JCI Insight. (2022) 7:e142843. doi: 10.1172/jci.insight.142843, PMID: 35132961 PMC8855814

[30] Xing Y Ren ZQ Jin R Liu L Pei JP Yu K. Therapeutic efficacy and mechanism of CD73-TGFβ dual-blockade in a mouse model of triple-negative breast cancer. Acta Pharmacol Sin. (2022) 43:2410–8. doi: 10.1038/s41401-021-00840-z, PMID: 35082394 PMC9433380

[31] Kong Y Jia B Zhao C Claxton DF Sharma A Annageldiyev C. Downregulation of CD73 associates with T cell exhaustion in AML patients. J Hematol Oncol. (2019) 12:40. doi: 10.1186/s13045-019-0728-3, PMID: 31014364 PMC6480867

[32] Tóth I le AQ Hartjen P Thomssen A Matzat V Lehmann C. Decreased frequency of CD73+CD8+ T cells of HIV-infected patients correlates with immune activation and T cell exhaustion. J Leukoc Biol. (2013) 94:551–61. doi: 10.1189/jlb.0113018, PMID: 23709688

[33] Panigrahi S Bazdar DA Albakri M Ferrari B Antonelli CJ Freeman ML. CD8+ CD73+ T cells in the tumor microenvironment of head and neck cancer patients are linked to diminished T cell infiltration and activation in tumor tissue. Eur J Immunol. (2020) 50:2055–66. doi: 10.1002/eji.202048626, PMID: 32548862

