# Supplementary figures and images for: CD73+CD8+ T cells define a subset with anti-tumor potential in DLBCL patients
Source: Front Med (Lausanne). 2025 May 2;12:1526772. doi: 10.3389/fmed.2025.1526772 (PMC12081347; doi:10.3389/fmed.2025.1526772)

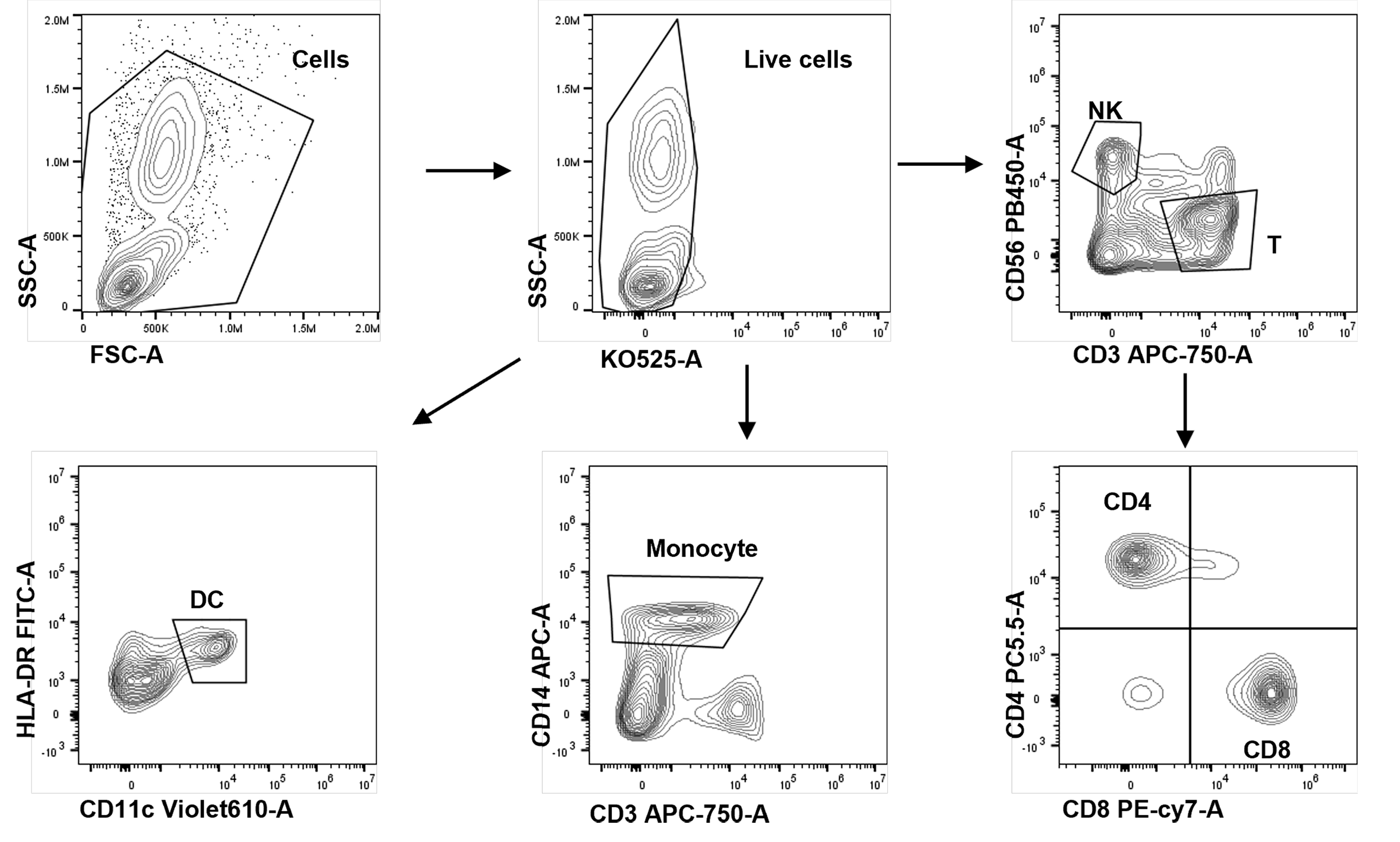

Supplement: Supplementary Figure S1 — The flow cytometric gating strategy for analyzing the proportions of live cells, CD4+ T cells, CD8+ T cells, NK cells, monocytes and DC. [file Image_1.tif]
